# Diagnosis of intravascular large B cell lymphoma: novel insights into clinicopathological features from 42 patients at a single institution over 20 years

**DOI:** 10.1111/bjh.16081

**Published:** 2019-07-03

**Authors:** Kosei Matsue, Yoshiaki Abe, Kentaro Narita, Hiroki Kobayashi, Akihiro Kitadate, Masami Takeuchi, Daisuke Miura, Kengo Takeuchi

**Affiliations:** ^1^ Division of Haematology/Oncology, Department of Internal Medicine Kameda Medical Centre Chiba Japan; ^2^ Division of Pathology The Cancer Institute, Japanese Foundation for Cancer Research Tokyo Japan

**Keywords:** intravascular large B‐cell lymphoma, random skin biopsy, bone marrow biopsy, PET‐CT, MRI

## Abstract

This study aimed to clarify the comprehensive clinical, laboratory, pathological and imaging features of intravascular large B‐cell lymphoma (IVLBCL) using data on 42 IVLBCL patients diagnosed at our hospital over the past 20 years. The majority of patients were diagnosed via random skin biopsy (29/42, 69·0%) followed by bone marrow biopsy alone (8/42, 19·0%). Characteristic features included persistent fever (41/42, 97·6%), decreased performance status (≥2) (100%), hypoxaemia (32/40, 80·0%), impaired consciousness (19/42, 45·2%), hypoalbuminemia (42/42, 100%) and extreme elevation of lactate dehydrogenase and soluble interleukin 2 receptor levels. Brain magnetic resonance imaging showed abnormal findings in 32/37 patients (86·4%). Hyperintense lesion in the pons was a peculiar finding that was unrelated to the neurological deficits. Positron emission tomography‐computed tomography revealed a high incidence of bone marrow (26/34, 76·5%), spleen (19/34, 55·9%) and adrenal gland (9/34, 26·5%) involvement. Neurolymphomatosis was noted in 6 patients during the course of the disease. About 60% of IVLBCL patients in whom *in vivo* diagnosis was possible survived more than 5 years with combination chemotherapy. Our observations provide additional insight into the diagnosis of IVLBCL and indicate that early disease recognition via random skin biopsy combined with imaging, enables *in vivo* diagnosis of the disease and improved survival for many patients.

Intravascular large B‐cell lymphoma (IVLBCL) is a distinct type of extranodal diffuse large B‐cell lymphoma (DLBCL) characterized by selective growth of lymphoma cells within the lumen of small blood vessels (Nakamura *et al*, 2008; Nakamura *et al*, 2017). IVLBCL usually affects elderly individuals and presents with various clinical symptoms, including persistent fever of unknown origin, altered consciousness levels, and unexplained hypoxaemia (Ferreri *et al*, 2004; Shimada *et al*, 2009). As lymphadenopathy is rare in patients with IVLBCL, the diagnosis of IVLBCL is often difficult and requires histological confirmation. IVLBCL is an aggressive form of DLBCL, and a delay in diagnosis often results in fatal outcomes. Biopsy of the affected organ, such as the lung, spleen or brain, is usually difficult because of the patient’s deteriorated condition.

We previously reported the usefulness of random skin biopsy (RSB) from normal‐appearing skin for the early diagnosis of IVLBCL (Asada *et al*, 2007; Matsue *et al*, 2011a). Given that patients are often suspected to have IVLBCL based on peculiar laboratory features, such as extremely elevated levels of serum lactate dehydrogenase (LDH) and soluble interleukin 2 receptor (sIL2R), in addition to the clinical symptoms, we have been using RSB for early diagnosis of IVLBCL for such patients with high sensitivity and specificity (Matsue *et al*, 2019).

Since Ferreri *et al *(2004) comprehensively described the clinical features of IVLBCL 15 years ago, the number of reported cases of IVLBCL has increased in both Asian (Pongpudpunth *et al*, 2015; Sitthinamsuwan *et al*, 2017) and Western (Cho *et al*, 2017) countries. However, due to the small sample size (Arai *et al*, 2016), heterogeneity of diagnostic modalities (Ha *et al*, 2014; Brunet *et al*, 2017), cross‐sectional design in the reported multi‐institutional studies (Ferreri *et al*, 2007; Murase *et al*, 2007; Shimada *et al*, 2009; Brunet *et al*, 2017) and early diagnosis by the use of RSB, we feel that the current studies reported in the literature do not depict an accurate clinical representation of IVLBCL.

This study aimed to clarify the comprehensive clinical and laboratory features of IVLBCL by analysing our clinical experiences of the diagnosis and clinical features for 42 IVLBCL patients examined at our hospital over the past 20 years. Although this study still used a small cohort, to the best of our knowledge, this work is the largest single institutional study of IVLBCL reported to date.

## Patients and methods

We retrospectively reviewed the records of patients who were diagnosed with IVLBCL according to the 2008 World Health Organization criteria (Nakamura *et al*, 2008) and its revision (Nakamura *et al*, 2017). Patient characteristics, including neurological abnormalities, laboratory data, including haematological and immunological findings, and biochemical examinations upon admission and thereafter were collected from electronic medical records from August 1999 to March 2019 at Kameda Medical Centre, Kamogawa‐shi, Chiba, Japan. Pathological data of all biopsied specimens, including the skin, lung and bone marrow were also obtained. Imaging reports of baseline whole‐body computed tomography (CT), brain magnetic resonance imaging (MRI), and positron emission tomography‐computed tomography (PET‐ CT) before the treatment were reviewed. Data of first‐line therapy and the clinical outcomes, including information on relapse and survivals, were recorded.

Biopsied specimens or autopsied organs were stained with haematoxylin‐eosin and immunostained using monoclonal antibodies against CD3, CD20, CD5, BCL2, BCL10, BCL6, MUM1 and CD31. Specimens obtained from bone marrow aspiration were examined by standard May‐Giemsa stain and flow‐cytometry to detect clonal B cells. Patients with prominent lymphadenopathy or massive tumour formation were excluded, even if IVLBCL lesions were observed in different organs. Except for the patients diagnosed with IVLBCL via autopsy, all patients underwent imaging studies upon admission, including whole body CT, brain MRI and PET‐ CT whenever possible. All patients, except those diagnosed before October 2006, who had symptoms indicative of IVLBCL (i.e. unexplained persistent fever, extremely elevated serum LDH and IL2R levels, and absence of prominent adenopathy) were evaluated by both RSB and bone marrow biopsy as an initial work‐up for the diagnosis of IVLBCL (Matsue *et al*, 2019). RSB was performed by the dermatology department and specimens were usually obtained from 3 separate, fat‐containing areas of the skin before the start of treatment. Given that intravascular IVLBCL lesions usually present in the capillaries of subcutaneous adipose tissues, the RSB sample was taken from the thigh, abdomen or upper arm, because these areas contain enough width and depth of subcutaneous adipose tissues (Asada *et al*, 2007; Matsue *et al*, 2011a; Sitthinamsuwan *et al*, 2017; Enzan *et al*, 2019).

Written informed consent for RSB was obtained from all patients or their family. The study was conducted in accordance with the Declaration of Helsinki and was approved by the Institutional Review Board of Kameda Medical Centre (Chiba, Japan).

### Statistical analysis

Progression‐free survival (PFS) was calculated from the date of diagnosis to documented disease relapse/progression or the date of death for patients who died from any cause. Overall survival (OS) was defined as the time between diagnosis and the date of last follow‐up or death. Survival curves were calculated using the Kaplan–Meier method and were compared using the log‐rank test.

## Results

### Patient characteristics

A total of 42 patients were diagnosed with IVLBCL. The baseline clinical features of patients at the time of diagnosis are presented in Table 1. RSB and bone marrow biopsy were performed in 36 patients as the initial work‐up within 3 days of admission. One patient received high dose steroid and 2 patients received low dose steroid (1 mg/kg/day, orally) for high fever; treatment was ceased in all 3 patients 3 days prior to receiving RSB. IVLBCL was diagnosed via RSB in 29 patients, bone marrow biopsy alone in 8, lung biopsy in 1, adrenal biopsy in 1 and autopsy in 3. Bone marrow lymphoma infiltration was detected in 24/40 patients (60·0%). All patients presented with significantly decreased general conditions (Eastern Cooperative Oncology Group performance score ≥2) and persistent fever of unknown origin. Neurological abnormalities, including various degrees of impaired consciousness, were the most frequently observed neurological abnormality, reported in 19/42 patients (45·2%). Heterogeneous focal neurological deficits were also reported; however, MRI abnormalities included ischaemic changes of brain lesions, which did not explain the neurological deficits observed in the patient in the majority of cases. Neurolymphomatosis was observed in 2 patients at the time of diagnosis, and indicated relapse another 4. Impaired consciousness was not recovered fully in 2 patients even after complete resolution of IVLBCL. The remaining 40 patients who responded favourably to rituximab and cyclophosphamide, dodorubicin, vincristine and prednisone (R‐CHOP) recovered fully without focal neurological deficit except for the 3 of the 4 patients with neurolymphomatosis.

**Table 1:** Clinical characteristics of the 42 patients with IVLBCL.

Clinical characteristics	Patients (*N* = 42)
Age, years; median (range)	73 (48–90)
Sex, male *n* (%)	22 (52·4)
*In vivo* diagnosis possible	39 (92·9)
Random skin biopsy *n* (%)	29 (69·0)
Bone marrow biopsy alone *n* (%)	8 (19·0)
Others *n* (%)	2 (4·8)
Symptoms	
Persistent fever *n* (%)	41 (97·6)
Impaired consciousness *n* (%)	19 (45·2)
Skin changes *n* (%)	6 (14·3)
ECOG PS ≥ 2 *n* (%)	42 (100)
Neurolymphomatosis *n* (%)	6 (14·3)
Laboratory findings	
Anaemia (Hb < 110 g/l) *n* (%)	29 (69·0)
Platelet count <120 × 10^9^/l *n* (%)	34 (81·0)
Saturation O_2_ ≤ 95% *n* (%)	32/40 (80·0)
Serum albumin, g/l; median (range)	22 (14–33)
LDH iu/l; median (range)	934 (412–4610)
sIL2R u/ml; median (range)	6886 (1920–32 412)
Ferritin μg/l; median (range)	1215 (109–15 920)
Fibrinogen <1·5 g/l *n* (%)	5 (11·9)
Triglyceride >3·0 mmol/l *n* (%)	4/40 (10·0)
Lymphoma cells in PB *n* (%)	5 (11·9)
Bone marrow histiocytosis^a^ *n* (%)	16 (38·1)
Bone marrow involvement^b^ *n* (%)	24/40 (60·0)
Chromosome abnormality *n* (%)	20/34 (58·8)
CD5‐positive *n* (%)	18/38 (47·4)

ECOG PS, Eastern Cooperative Oncology Group performance status; Hb, haemoglobin; IVLBCL, intravascular large B‐cell lymphoma; LDH, lactate dehydrogenase; PB, peripheral blood; sIL2R, soluble interleukin 2 receptor.

a Assessed by bone marrow smear

b Assessed by bone marrow biopsy

Anaemia, thrombocytopenia and unexplained hypoxaemia were the most frequently observed laboratory abnormalities. All patients exhibited extremely low levels of serum albumin, high levels of serum LDH, sIL2R and ferritin. Lymphoma cell infiltration in the peripheral blood and bone marrow were observed in 11·9% and 60·0%, respectively. Various degrees of bone marrow histiocytosis (histiocytosis with 1–10 haemophagocytes per 500 nucleated cells) was observed in 16/42 patients (38·1%).

Chromosomal abnormalities were detected in 20 bone marrow samples from the 34 patients in whom karyotype analysis was successful. Among them, bone marrow lymphoma infiltration was not detected in 4 patients (Table S1). A common pattern of abnormality could not be defined due to the heterogeneous and complex karyotype pattern of the disease and the small sample size.

### Imaging studies

Baseline imaging studies included whole body CT scan, brain MRI and PET‐CT in 42, 37 and 34 patients, respectively (Table 2). One patient received high‐dose steroid for 2 weeks before being admitted. Imaging findings are summarized in Table 2. Prominent lymphadenopathy was not detected in all patients, but small intrapleural or intraperitoneal lymphadenopathy (<2 cm in diameter) was often observed. Splenomegaly and adrenal tumours were detected in 34/42 (81·0%) and 11/42 (26·2%) patients, respectively. Chest CT detected pleural effusion in 15/42 (40·0%) and ground glass opacity of lung field in 9/42 (21·4%) patients.

**Table 2:** Imaging studies of patients with IVLBCL.

Imaging study	Findings	*N* (%)
CT performed		42 (100)
	Splenomegaly	36 (85·7)
	Adrenal tumour	11 (26·2)
	Pleural effusion	15 (35·7)
	GGO	10 (23·8)
Brain MRI performed		37 (100)
	Abnormal	32 (86·4)
	Hyperintense lesion in the pons on T2WI	20 (54·1)
	Nonspecific white matter lesions	17 (45·9)
	Infarct‐like lesions	10 (27·0)
	Meningeal enhancement	4 (10·8)
PET‐CT performed		34 (100)
	Bone marrow FDG‐uptake (+)	24 (70·5)
	Focal	8 (23·5)
	Diffuse	16 (47·1)
	Splenomegaly with FDG uptake	19 (55·9)
	Adrenal gland uptake	9 (26·5)
	Lymph node uptake	9 (26·5)
	No uptake	10 (29·4)

CT, computed tomography; FDG, ^18^F‐fluorodeoxyglucose; GGO, ground glass appearance; IVLBCL, intravascular large B‐cell lymphoma; MRI, magnetic resonance imaging; PET, positron emission tomography

Brain MRI abnormalities were detected in 32/37 (86·2%) patients, with hyperintense lesions in the pons in 20/37 (54·1%), non‐specific white matter lesions in 17/37 (45·9%), infarct‐like lesions in 10/37 (27·0%) and meningeal enhancement in 4/37 (10·8%) patients.

**Figure 1:**
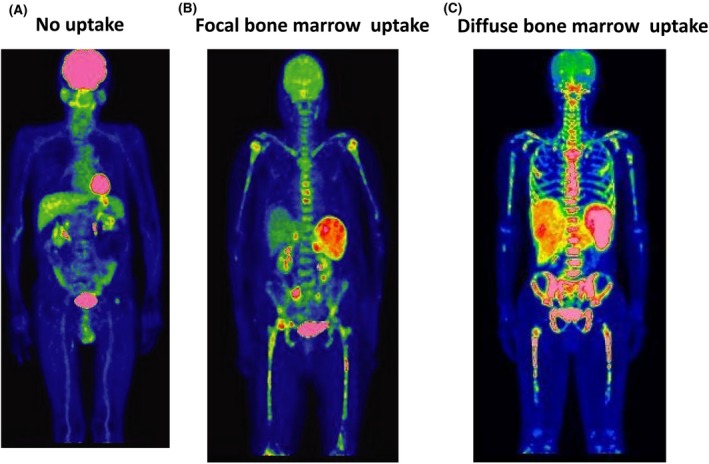
Bone marrow accumulation pattern of FDG in patients with IVLBCL. Bone marrow accumulation of fluorodeoxyglucose (FDG) was divided into three patterns*;* no uptake (A), focal (B) and diffuse (C), according to the positivity of uptake in central and appendicular bones

Lymphoma infiltration of the bone marrow was assessed by PET‐CT in 34/42 patients, and was reported as positive in 24/34 patients (70·6%). Bone marrow infiltration was considered positive if fluorodeoxyglucose (FDG)‐avid lesions in the bone marrow were more intense than those in the liver, and disappeared concomitantly with the disappearance of other lymphoma lesions on PET‐CT monitoring (Berthet *et al*, 2013). Bone marrow accumulations were divided into 3 patterns: no uptake (*n* = 10), focal bone marrow uptake (*n* = 8) and diffuse bone marrow uptake (*n* = 16) (Fig 1).

When comparing data on lymphoma infiltration by bone marrow biopsy and by PET‐CT in 34 patients, 18 patients were reported positive on both modalities, while 7 patients were found to be negative on both modalities. There were 6 and 3 patients who were PET‐CT‐positive/biopsy‐negative and PET‐CT‐negative/biopsy‐positive, respectively.

Splenomegaly was observed in 29 of 34 (85·3%) patients with PET‐CT data, but increased splenic uptake was observed only in 19/34 (55·9%) patients, with standard uptake value ranging from 2·91 to 8·23. Two patients showed bilateral FDG‐uptake by the renal cortex, which was shown to be an infiltration of IVLBCL after renal biopsy (Kimura *et al*, 2017). Nine of 34 patients (26·5%) with adrenal enlargement exhibited significantly abnormal FDG‐uptake on PET‐CT. Other findings included diffuse weak lung accumulation in 2 patients and intrapleural or intraperitoneal lymph node accumulations in 8 patients (23·5%). Four patients did not show FDG accumulation in any organs, including the skin.

### Pathological features

Three patients were diagnosed post‐mortem by autopsy. IVLBCL lesions were found in almost all organs including the thyroid, liver, pancreas, prostate and brain. As these 3 patients did not receive any treatment for lymphoma, massive infiltrations outside the vessels were also observed.

All specimens obtained by RSBs contained sufficient subdermal adipose tissues. Specimens with IVLBCL lesions in the dermis always contained more IVLBCL lesions in the subdermal adipose tissue but the reverse was not true (Figure S1). Skin change was noted in 6 patients, but no clear correlation between the presence or absence of cutaneous lesions and IVLBCL lesions was noted.

**Figure 2:**
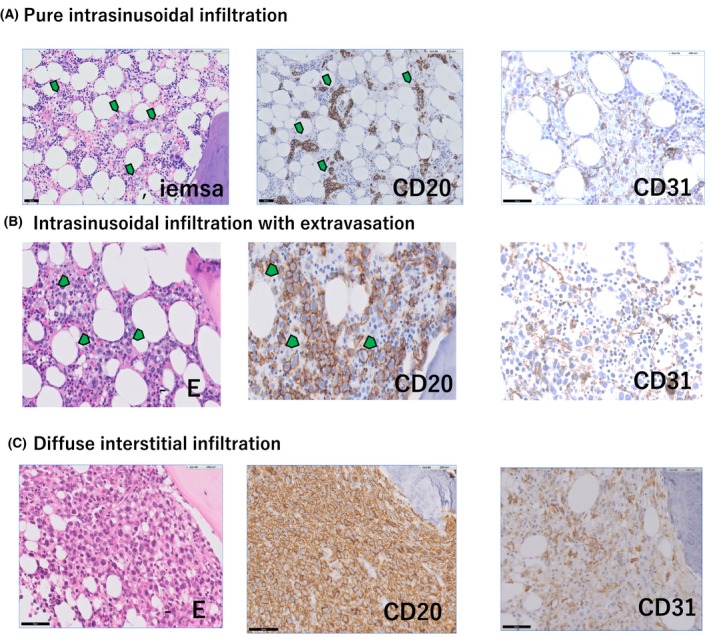
Bone marrow infiltration pattern in patients with IVLBCL. (A) Pure intrasinusoidal infiltration. Neoplastic cells are confined within the intrasinusoidal spaces depicted by CD31 immunostaining. Green arrows indicate lymphoma cells. (B) Intrasinusoidal infiltration with extravasation. Neoplastic cells proliferate within the intrasinusoidal space but extravasation was also seen. (C) Diffuse interstitial infiltration pattern. Neoplastic cells proliferate diffusely within the bone marrow

IVLBCL was diagnosed by bone marrow biopsy alone in 8 patients. Concerning the involvement of the bone marrow in IVLBCL, intrasinusoidal infiltration of lymphoma cells was typically observed, which was then confirmed through immunostaining with CD31 monoclonal antibody, although various degrees of extravasation was often observed (Fig 2). Paratrabecular infiltration pattern was not observed. We excluded the patients with massive nodular or interstitial infiltration pattern, in whom definitive IVLBCL lesions were not confirmed in sites other than the bone marrow. CD5 was positive in 18/38 patients (47·7%). Unlike previous reports (Murase *et al*, 2007; Brunet *et al*, 2017), we did not find any patient with CD10‐positive staining in our cohort.

### Treatment

Three patients did not receive chemotherapy for lymphoma as they were diagnosed post‐mortem. Two patients refused treatment. All of them were treated with various doses of steroid. The remaining 37 patients received at least 2‐cycles of R‐CHOP (median, 6 cycles) followed by 7–15 mg intrathecal methotrexate (MTX) for each course. Although rituximab (375 mg/m^2^) was administered at full dose, the doses of other cytotoxic agents (including intrathecal MTX) were reduced by the attending physicians according to the patient’s general condition, comorbidities and age. High‐dose MTX (1·0–3·5 g/m^2^) was added after 2 and 4 cycles of R‐CHOP for patients diagnosed after 2010, and whenever feasible (patients without adequate renal function, estimated glomerular filtration rate <30 ml per hour or age ≥80 years were excluded). Two patients who had neurolymphomatosis of the cauda equina at presentation also underwent involved field irradiation. Another 2 patients received haematopoietic stem cell transplantation (1 autologous and 1 allogeneic).

### Outcomes

The median follow‐up time was 17·9 months (range; 0·4–209 months), the median OS was 105 months and the 5‐year survival rate was 53·0% [95% confidence interval (CI): 32·5–65·8%]. Moreover, the median OS of patients who received chemotherapy was 135·3 months with a 5‐year survival rate of 62·0% (95% CI: 39·4–74·6%). No difference in survival was observed between the patients with or without CD5‐positivity (Figure S2). In total, 19 of the 42 patients died during the observation period. Five patients who did not receive lymphoma treatment due to post‐mortem diagnosis (*n* = 3) and refusal of treatment (*n* = 2) died between 0·7 and 22 months. Of note, 3 of these patients survived more than 1 year with palliative steroid treatment and supportive care. In the remaining 14 patients, 9 died due to lymphoma‐related causes, namely, induction failure in 5 and lymphoma relapse in 4. Among the 37 patients who received R‐CHOP, 17 patients received at least 1–2 courses of HD‐MTX and 2–6 courses of intrathecal MTX injection. The rest of the patients received intrathecal MTX alone. Relapse in brain parenchyma occurred only in 1 patient who received 4 courses of HD‐MTX and 6 courses of intrathecal MTX.

Four patients relapsed with neurolymphomatosis, as previously described and disease relapse in the brain was observed in only 1 patient, who received 2 courses of HD‐MTX. None of the patients who did not receive HD‐MTX had CNS relapse. Five patients died from non‐lymphoma related causes including aspiration pneumonia in 3, renal failure in 1 and neuroendocrine cancer in 1.

## Discussion

This study is the largest series of IVLBCL patients examined, diagnosed and treated at a single institution over a 20‐year period. We performed a comprehensive work‐up including bone marrow biopsy, RSB, whole body CT, brain MRI and PET‐CT, in addition to routine laboratory examinations in patients suspected of IVLBCL. Pathological analysis included immunohistochemical staining using various monoclonal antibodies. Despite the long study duration, all the patients with *in vivo* diagnosis, except one, were uniformly treated with rituximab and CHOP, although the doses of cytotoxic agents were modified according to patient condition. The 5‐year survival rate of treated patients was approximately 60%, and this value was better than the 5‐year survival rate of 46·4% from the Surveillance, Epidemiology, and End Results (SEER) database from the USA (Rajyaguru *et al*, 2017).

The clinical and laboratory features of the current study were compared with the previous largest series from Japan and Western countries (Table 3). Our finding is very different to that reported in both these studies (Murase *et al*, 2007; Ferreri *et al*, 2007). Most patients were diagnosed by bone marrow biopsy (67%) in the Japanese series (Murase *et al*, 2007) and by skin biopsy from positive skin lesions (38%) in the Western series (Ferreri *et al*, 2007), while we used RSB as our diagnostic method of choice.

**Table 3:** Clinical and laboratory features in previous Japanese cases, Western cases and current report.

	Japanese cases^a^	Western cases^b^	Current report
Number of patients	*N* = 96	*N* = 50	*N* = 42
Median age, years	67	68	73
Main diagnostic site			
Bone marrow	67	15	19
Skin	16	38	69
Fever	87	42	98
Neurological symptoms	27	42	45
Hypoxaemia	34	18	80
Skin eruptions	15	38	14
Anaemia (Hb < 110 g/l)	66	66	95
Thrombocytopenia (10 x10*g/l)	58	32	69
Hypoalbuminaemia (30 g/l)	47	NR	86
Bone marrow histiocytosis	61	0	38
LDH > ULN	93	85	100
sIL2R > 5000 u/ml	56	NR	69
Splenomegaly	67	26	81
Bone marrow involvement	74	30	60
Peripheral blood involvement	24	4	14

Values are expressed as a percentage of patients. LDH, lactate dehydrogenase; NR, not reported; sIL2R, soluble interleukin 2 receptor; ULN, upper limit of normal.

a Murase *et al *(2007).

b Ferreri *et al *(2007).

After reporting on the usefulness of RSB (Asada *et al*, 2007), 29 of 39 patients (74·4%) were diagnosed via this method and only 8 patients (20·5%) were diagnosed via bone marrow biopsy alone. RSB is now considered as a standard diagnostic procedure for patients with suspected IVLBCL in Japan but not in Western countries. One important reason that RSB has not been widely used in Western countries might be its limited diagnostic capability for IVLBCL, probably because those samples are collected by punch biopsy (Gill *et al*, 2003; Cho *et al*, 2017), which, despite being easy to perform, might not contain sufficient amounts of hypodermal adipose tissue for diagnosis. We recently reported the low frequency of IVLBCL lesions in the dermis and showed that most IVLBCL lesions can be found mainly in the hypodermic adipose tissues (Enzan *et al*, 2019). Hence, punch biopsy might miss the IVLBCL lesion in approximately more than half of the patients.

In our study, 8 patients were diagnosed via bone marrow biopsy alone. However, the diagnosis of IVLBCL by bone marrow biopsy alone is often problematic (Kajiura *et al*, 2007; Das *et al*, 2011), because the bone marrow sinusoids blend seamlessly with other haematopoietic tissues. In addition, the difference between bone marrow involvement of splenic DLBCL (Morice *et al*, 2005; Ponzoni & Ferreri, 2006; Iannitto & Tripodo, 2011; Shimono *et al*, 2017) and primary bone marrow DLBCL (Martinez *et al*, 2012) is not clearly defined. Splenic involvement has been traditionally determined by the enlargement of non‐functioning imaging studies, such as CT or abdominal ultrasound. In this study, PET‐CT revealed inconsistency between splenic enlargement and FDG‐uptake in 10 of 29 patients who presented with splenomegaly.

Although not always diagnostic, an intrasinusoidal infiltration pattern in the bone marrow is a hallmark characteristic for the diagnosis of IVLBCL, but the extravasation and nodular to interstitial infiltration patterns frequently coexisted even in the presence of IVLBCL lesions in the skin (Fig 2B, 2). As neoplastic cells of IVLBCL selectively proliferate within the lumina of capillaries outside the bone marrow, the proliferation patterns in the bone marrow and other organs, including the skin, might be different. Alternatively, bone marrow *per se* might be considered as a bundle of small capillaries, resulting in a unique pattern, which might not match the patterns observed in other organs. Furthermore, IVLBCL, splenic DLBCL, and primary bone marrow DLBCL often share common clinical, laboratory and immunophenotypic features, including unexplained fever, absence of prominent lymphadenopathy, splenomegaly, high levels of serum LDH and sIL2R, and CD5 positivity. These lymphomas might be grouped as a variant of extranodal DLBCL.

Imaging studies can aid in the diagnosis of IVLBCL. In this study, we first clarified several characteristic features of IVLBCL in a relatively large cohort of consecutive patients using multiple imaging modalities. As reported by our recent study (Abe *et al*, 2018), brain MRI abnormalities were detected in 86·2% of patients in this update and hyperintense lesion in the pons were detected on T2‐weighted imaging, which was a peculiar finding observed in 54·1% of patients and unrelated to the impaired neurological functions. Pathological findings of these hyper‐intense lesions in the pons were previously described in a patient who underwent autopsy for vascular occlusion of small vessels (Yamamoto *et al*, 2012).

Although PET‐CT did not detect IVLBCL skin lesions, it did detect the lesions in the bone marrow (76·6%) and adrenals (26·5%) with higher frequency than that reported in a previous Japanese study (Murase *et al*, 2007) and Western case series (Ferreri *et al*, 2007). Splenomegaly is a common finding on both CT (81·0%, 34/42) and PET‐CT (85·3%, 29/34), but increased splenic uptake of FDG was observed in 19 of 34 patients (57·6%) with splenomegaly on baseline PET‐CT.

Recently, Suehara *et al* (2018) reported the usefulness of liquid biopsy for identification of IVLBCL using *MYD88* and *CD79B* Y196 mutations. The high positivity of tumourous DNA by liquid biopsy could also be explained by the high prevalence of bone marrow involvement as well as intravascular localization of lymphoma cells.

In contrast to previous studies (Murase *et al*, 2007; Shimada *et al*, 2008), and despite the high frequency of neurological abnormalities and abnormal findings on brain MRI, CNS recurrence was not frequent in our study. No CNS relapse was seen, even in the 18 patients who did not receive HD‐MTX. Given that most abnormal findings detected on MRI appeared to relate to ischaemic changes of small brain capillaries due to neoplastic lymphoma cells, and all neurological symptoms disappeared after treatment in most patients, there is the possibility that most neurological abnormalities observed in IVLBCL might be associated with occlusion of small vessels of the brain. As the patients who received R‐CHOP had low CNS relapse rate irrespective of HD‐MTX administration in our study, it might be possible that parenchymal infiltration might be infrequent in IVLBCL.

Neurolymphomatosis is an extremely rare disease, but it is a relatively frequent complication in patients with IVLBCL. We confirmed and extended our previous observation on the association of neurolymphomatosis and IVLBCL (Matsue *et al*, 2011b) in this study. Interestingly, 5 of the 6 patients with neurolymphomatosis in our cohort were positive for CD5.

Cutaneous involvement at diagnosis, an important geographic difference between the patients in Asian and European countries, was low in our cohort. Skin changes were observed in only 6 patients (15·4%) and were not related to the presence of IVLBCL lesions on RSB. It is possible that most IVLBCL lesions were in the hypodermic adipose tissue; therefore, those lesions may not develop clinically detectable skin changes in our patients. Most of these lesions in Western countries were reportedly located in the dermis (Roglin & Boer, 2007; Garcia‐Munoz *et al*, 2014; Nguyen *et al*, 2015), and the presence or absence of IVLBCL lesions in the hypodermic adipose tissues of normal appearing skin in other sites has not been mentioned in the reports of cutaneous variant of IVLBCL.

Bone marrow histiocytosis was less frequent in our study, in contrast to a previous study (38% vs. 61%) (Murase *et al*, 2000). Although not statistically significant, patients with bone marrow histiocytosis tended to have higher frequency of neurological abnormalities compared to those without [62·5% (10/16) vs. 30·4% (7/23), *P* = 0·1146]. The reason for these differences remains unclear, but most patients in reported previous studies may not have been diagnosed via RSB at an early stage of the disease. Although IVLBCL may trigger lymphoma‐associated haemophagocytic syndrome, we hypothesize that early diagnosis of the disease via RSB might be associated with the low frequency of bone marrow histiocytosis in our patients.

While our study has several limitations, including the retrospective, single institutional design of the study and not using novel molecular technique, it is strengthened by the fact that most the patients received an extensive clinical work‐up and were diagnosed comprehensively via RSB, bone marrow biopsy and advanced imaging methods such as MRI and PET‐CT at baseline. All patients were clinically suspected of and diagnosed with IVLBCL by 2 experts and received long‐term follow‐up at our hospital. Furthermore, to the best of our knowledge, this is the largest reported cohort of IVLBCL cases from a single institution. In addition, our observations explain some geographic differences between Western countries and Japan.

In conclusion, we described a comprehensive approach for the diagnosis and work‐up of patients with IVLBCL. We also described peculiar clinical and laboratory features of this disease, some of which were not consistent with the features of IVLBCL in the previous cross‐sectional multi‐institutional studies reported from Western countries and Japan.

We believe this study provides valuable observations for understanding the clinical and biological features of IVLBCL, and aids the early diagnosis of IVLBCL, which will increase the likelihood of providing appropriate effective treatments and ultimately lead to better survival for these patients.

## Author contributions

KM designed the study, collected the data and wrote the manuscript. KM, YA, KN, HK, AK, MT and DM managed the patients. YA performed the statistical analysis. KT reviewed the pathological diagnosis of the patients. All authors have reviewed and approved the manuscript.

## Conflict of interest

All authors have no conflict of interest to declare.

## Supporting information


**Figure S1.** Representative microscopic image of incisional random skin biopsy in a patient with IVLBCL.
**Figure S2.** Overall survival of patients with IVLBCL.
**Table S1.** Cytogenetic abnormalities of the 42 patients with IVLBCL.
