# Supplementary material for: Diagnosis of intravascular large B cell lymphoma: novel insights into clinicopathological features from 42 patients at a single institution over 20 years
Source: Br J Haematol. 2019 Jul 3;187(3):328–36. doi: 10.1111/bjh.16081 (PMC6900202; doi:10.1111/bjh.16081)
Supplement: Supplementary file 1 — Figure S1. Representative microscopic image of incisional random skin biopsy in a patient with IVLBCL. Figure S2. Overall survival of patients with IVLBCL. Table S1. Cytogenetic abnormalities of the 42 patients with IVLBCL. [file BJH-187-328-s001.docx]

**Supplemental Figures**

**Figure S1,** Representative microscopic image of incisional random skin biopsy in a patient with IVLBCL.

No IVLBCL lesion was found in the dermis, but many IVLBCL lesions were seen in the hypodermic adipose tissue. Square areas were zoomed and CD20 immunostaining was performed

**Figure S2, Overall survival of patients with IVLBCL**

**Table SI, Cytogenetic abnormalities of the 42 patients with IVLBCL**

**
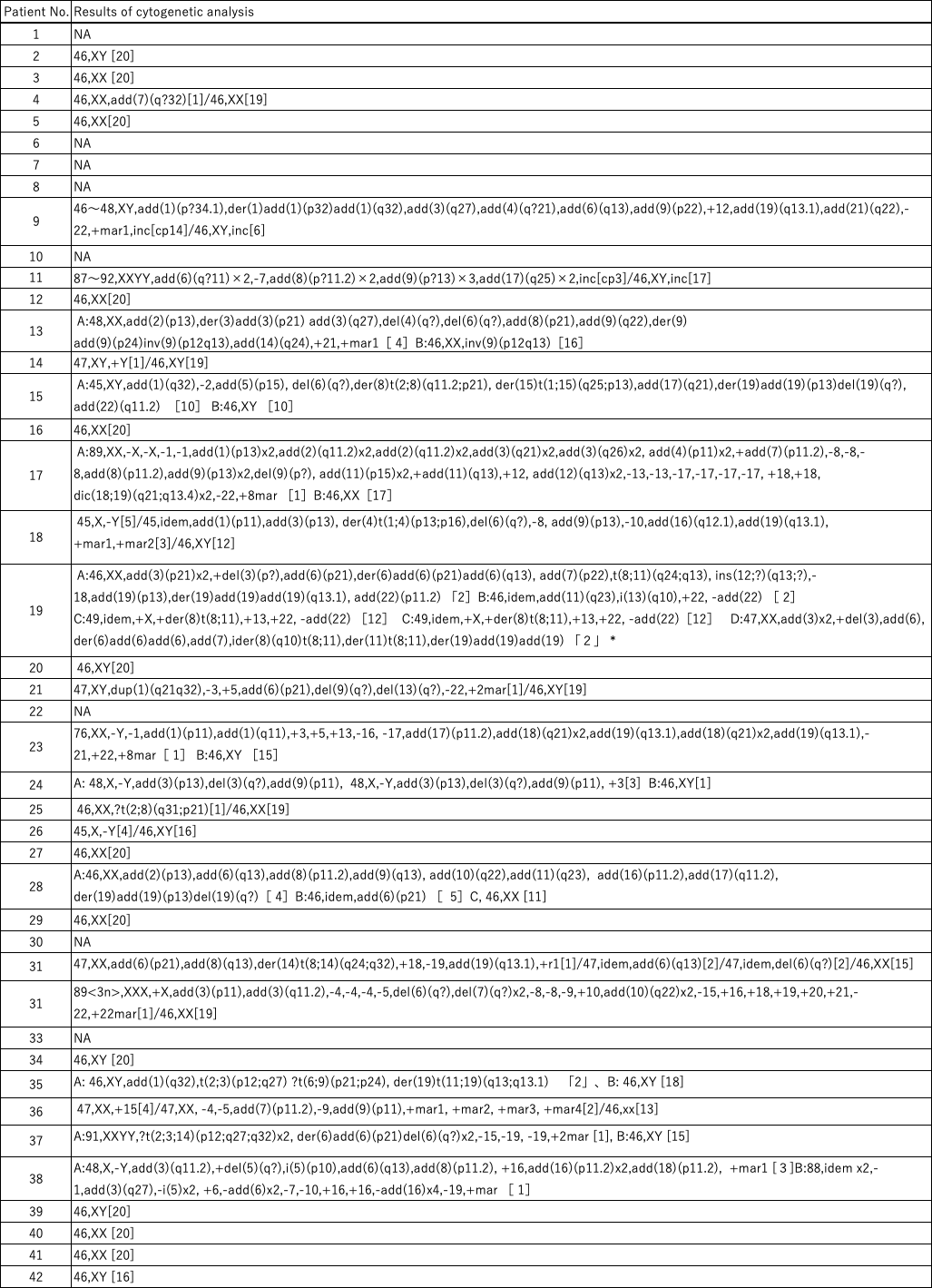
**
